# Recent Advances of Azobenzene-Based Photoresponsive Molecular Switches for Protein-Targeted Photopharmacology

**DOI:** 10.3390/molecules31071205

**Published:** 2026-04-05

**Authors:** Jingyu Jiang, Xinrui Yuan, Lei Hu

**Affiliations:** School of Pharmacy, Jiangsu University, Zhenjiang 212013, China; 2222315004@stmail.ujs.edu.cn

**Keywords:** azobenzene, photoswitch, stimuli-responsive, photopharmacology, prodrug

## Abstract

Azobenzene derivatives constitute a prototypical class of photoresponsive molecular switches with broad utility in synthetic chemistry and biomedical research, owing to their distinctive physicochemical properties. Recent molecular engineering has enabled red-shifted photoisomerization into the visible biological window, thereby enhancing tissue penetration and reducing phototoxicity. This review systematically surveys contemporary advances in azobenzene-based photoswitchable systems with a specific focus on medicinal chemistry and photopharmacology. Emphasis is placed on rational design strategies—including *ortho*-functionalization, heteroaryl substitution, and bridged diazocine scaffolds—that improve photophysical properties, thermal stability, and photostationary state distributions. Particular attention is devoted to the integration of these novel azobenzene motifs as privileged pharmacophores, highlighting their emerging therapeutic applications in neurological modulation, enzyme inhibition, receptor targeting, and oncology, as well as their translational potential in drug discovery and photodynamic therapy.

## 1. Introduction

Spatiotemporally precise regulation of drug activity is a long-standing core goal in medicinal chemistry, as it holds the key to reducing off-target toxicity and improving the therapeutic window of clinical agents. Photopharmacology, which uses light to modulate the bioactivity of drug molecules, has emerged as a promising strategy to achieve this goal, and azobenzene stands out as the most widely used and well-characterized photoresponsive molecular switch in this field.

**Figure 1:**
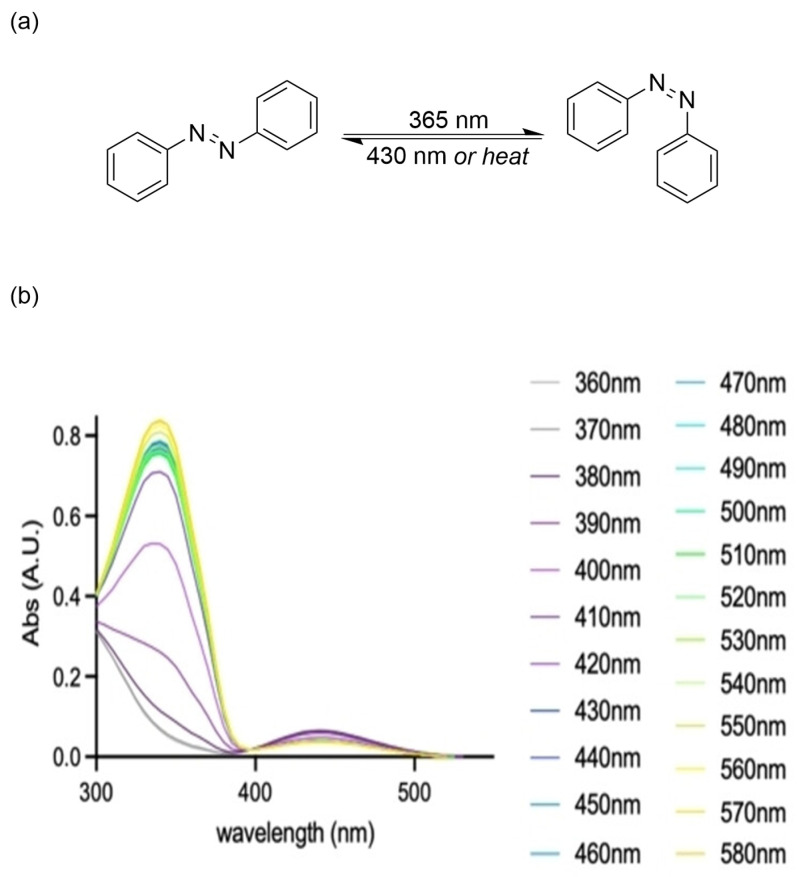
(**a**) Isomerization of azobenzene. (**b**) UV-Vis absorption spectra at distinct PSS. (**b**) Reproduced with permission from Ref. [12]. Copyright (2024) Wiley Online Library.

Azobenzene is a prototypical photoresponsive scaffold comprising two conjugated aromatic rings connected by an azo bond (-N=N-), which exists in two stereoisomeric states with distinct properties [1,2,3]. The thermodynamically stable *trans* isomer has a planar π-conjugated structure with extended electron delocalization, while the metastable *cis* isomer adopts a non-planar configuration, featuring disrupted conjugation and perpendicular dipole orientation. The reversible *trans ↔ cis* isomerization of azobenzene is driven by wavelength-specific light irradiation: *trans-to-cis* conversion is predominantly induced by ultraviolet light (typically ~365 nm) via excitation of the high-energy π → π* transition, while *cis-to-trans* reversion can be achieved either by irradiation with blue light (activating the lower-energy n → π* transition) or through thermal relaxation in the dark (Figure 1a) [1,4,5,6,7,8,9,10,11]. This typical bistable characteristic enables precise optical control over the molecular configuration and properties of suitable azobenzene derivatives. Two key parameters define the practical performance of azobenzene photoswitches: the photostationary state (PSS), a dynamic equilibrium in the photochemical system where the formation and disappearance rates of isomers are balanced, with different irradiation wavelengths leading to distinct PSS and corresponding characteristic UV-Vis absorption profiles (Figure 1b) [12]; and the half-life (t_1/2_), the time required for half of the metastable *cis* isomer to revert to the stable *trans* isomer via thermal relaxation. An ideal PSS distribution and sufficiently long half-life are core prerequisites for azobenzene switches to achieve robust and durable property regulation before and after photoactivation.

The photoregulation of molecular conformation forms the physical basis for using azobenzene as a molecular switch. Owing to these favorable features, azobenzene has been extensively investigated and applied in numerous fields, including materials chemistry, catalytic chemistry, supramolecular chemistry, and medicinal chemistry [13,14,15,16,17,18,19,20,21,22,23,24,25]. As a photo-controlled switch in bioactive molecules, azobenzene enables direct modulation of conformational changes and donor–acceptor interactions, thus exhibiting important research value in biomedical applications. Notably, the first azobenzene-based clinical candidate (KIO-301) developed by Kiora Pharmaceuticals is currently under clinical trials for vision restoration in patients with retinitis pigmentosa, further highlighting the translational potential of azobenzene photopharmacology.

However, conventional unmodified azobenzene systems face inherent bottlenecks that limit their clinical translation: the requirement of UV light for activation suffers from poor tissue penetration and potential phototoxicity, while suboptimal PSS ratios and insufficient thermal stability of the *cis* isomer further compromise the efficiency and durability of photoregulation [9,26]. To address these limitations, a series of structural engineering strategies for azobenzene have been developed in recent years, including *ortho*-functionalization, heteroaryl substitution, bridged diazocine construction, and macrocyclic design, which have realized red-shifted activation to the visible window, enhanced thermal stability, and improved switching performance [27,28,29,30,31,32,33].

While existing reviews have summarized the basic photochemical properties and general applications of azobenzene, few systematically integrate its structural optimization strategies with applications across multiple medicinal chemistry fields. To fill this gap, this review examines recent progress in azobenzene photoswitches within medicinal chemistry and chemical biology. We highlight strategies to red-shift activation into the visible window and to enhance thermal stability through *ortho*-functionalization, heteroaryl substitution, and bridged diazocine scaffolds. Representative photopharmacological applications include light-gated control of ion channels (Kv, GIRK, TRPA1), glycosidase inhibition, adrenergic (α_2_/β_2_) and muscarinic receptor modulation, cholinesterase inhibition, and oncology targets (proteasome, VEGFR2, tubulin). This review aims to establish a clear structure-property-activity relationship for azobenzene in medicinal chemistry, and provide a practical reference for the development of novel light-regulated therapeutic agents.

## 2. Photoresponse of Azobenzene

### 2.1. Photoresponse of Linear Azobenzene

**Figure 2:**
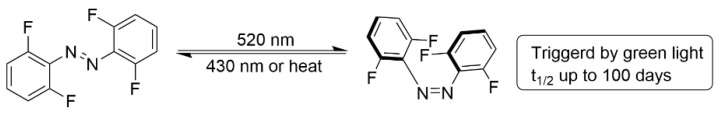
Tetra-*ortho*-fluorination prolonged azobenzene half-life and altered its isomerization response wavelength.

With in-depth investigations in recent years, researchers have developed various methods to adjust the π → π* transition energy of the azobenzene bond to regulate the light-responsive characteristics of the azobenzene structure. Systematic *ortho*-fluorination represents a landmark strategy [10,28,34]. *Ortho*-fluorinated azobenzenes were first described by Bléger and Hecht, who established a visible-light-responsive photochromic platform based on tetra-*ortho*-fluorinated azobenzene derivatives [35]. Notably, tetra-*ortho*-fluorinated azobenzenes show a significantly red-shifted excitation wavelength and prolonged *cis* isomer thermal half-life relative to the parent azobenzene. This is attributed to the electron-withdrawing effect of *ortho*-fluorine substituents, which stabilize the lone pair electrons of the azo bond, lower the n orbital energy level, and achieve spectral separation of the n−π* absorption bands of the two isomers. Feringa and his colleagues further investigated the photo-responsive properties of *ortho*-fluorinated azobenzene compounds [36]. As shown in Figure 2, tetra-*ortho*-fluorination red-shifted the excitation wavelength by 155 nm (λ = 520 nm vs. 365 nm for parent azobenzene), enabling visible-light photoswitching while extending *cis*-isomer thermal stability (t_1/2_ > 100 days at 25 °C). The engineered fluorinated system exhibited enhanced photoisomerization quantum yield within the visible spectral window, demonstrating significant advantages over conventional UV-activated analogues in biological compatibility and operational persistence.

**Figure 3:**
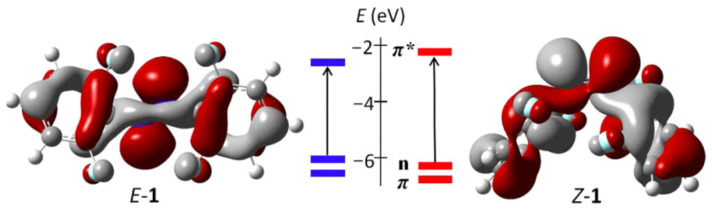
Energetic diagram of the π, n, and π* orbitals of *ortho*-fluorinated azobenzene, and representation of the n-orbitals (HOMOs) calculated at the B3LYP/6-31G(d) level of theory (arrows highlight n → π* transitions). Reproduced with permission from Ref. [35]. Copyright (2012) American Chemical Society.

With the rapid advancement of computational science and technology, theoretical methods including density functional theory (DFT) simulations and machine learning approaches have been increasingly employed in azobenzene-related research, facilitating in-depth exploration of its photophysical and photochemical properties. [22,37,38,39,40,41,42].These methods offer a quantitative framework for understanding the photoreponsive properties of azobenzene and the mechanisms underlying the effects of diverse structural modifications. The photochemical behavior of *ortho*-fluorinated azobenzene has also been mechanistically elucidated through orbital engineering principles [43,44,45,46]. Hecht and co-workers revealed that fluorination proximal to the azobenzene moiety induces n-orbital destabilization in the *cis*-isomer (Figure 3), resulting in energetic decoupling of n→π* transitions between *trans/cis* configurations [35]. This electronic modulation conferred exceptional thermal resilience to the *cis*-isomer (t_1/2_ exceeding 2 years at 25 °C) through hyperconjugative interactions between fluorine lone pairs and the π-system. Quantum chemical analyses further showed that *ortho*-fluorine substituents exert dual electron-withdrawing (inductive) and steric effects, distorting the azo bond and amplifying differences in n → π* oscillator strength between isomers. These synergistic electronic and conformational effects collectively enhance *cis*-isomer population retention in PSS (PSS_cis_ > 85%) while suppressing thermal relaxation pathways via non-covalent F···H–C interactions that stabilize the *cis*-isomer ground state and raise the thermal isomerization barrier.

**Figure 4:**
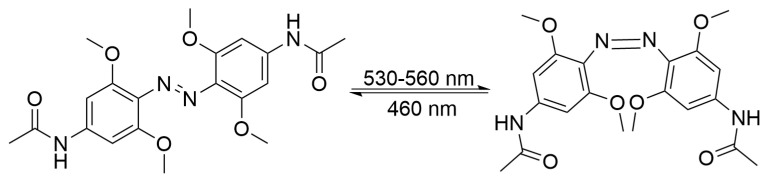
The isomerization process of tetra-*ortho*-methoxy-substituted azobenzenes.

In addition, apart from tetra-*ortho*-fluoro-substituted azobenzenes, other tetra-*ortho*-substituted derivatives (e.g., methoxy- and chloro-substituted azobenzenes) can also be activated by visible light, thus expanding the strategies available for improving their biocompatibility. In a series of imide-based azobenzene derivatives reported by Woolley’s group, four *ortho* positions were fully substituted with methoxy groups, leading to a remarkable red shift of the n → π* absorption band of the *trans* isomer [47]. As shown in Figure 4, the *trans-to-cis* isomerization could be triggered upon green-light irradiation (530–560 nm), while the reverse *cis-to-trans* conversion was achieved using blue light (460 nm). The *cis* isomer exhibited a half-life of approximately 2.4 days in aqueous solution in the dark, realizing thermally stable, bidirectional photoisomerization.

**Figure 5:**
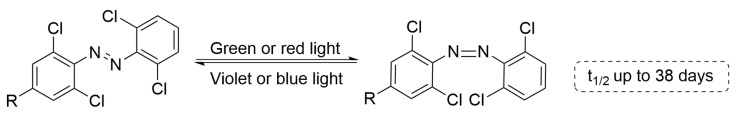
The isomerization process of tetra-*ortho*-chloroazobenzenes.

Jacquemin and co-workers systematically characterized the photochromic properties of tetra-*ortho*-chloroazobenzenes [48]. As shown in Figure 5, for derivatives with different substituents on the tetra-*ortho*-chloroazobenzenes framework, the *trans*-to-*cis* isomerization could be typically triggered by green light at 526 nm or red light at 625 nm, while the reverse *cis*-to-*trans* isomerization was mainly induced by irradiation with 426 nm blue-violet light. The excitation wavelength redshift correlated with the presence of S_0_–S_1_ absorption bands, which were conventionally assigned to n–π* transitions.

### 2.2. Photoresponse of Heteroaryl Azobenzene

**Figure 6:**
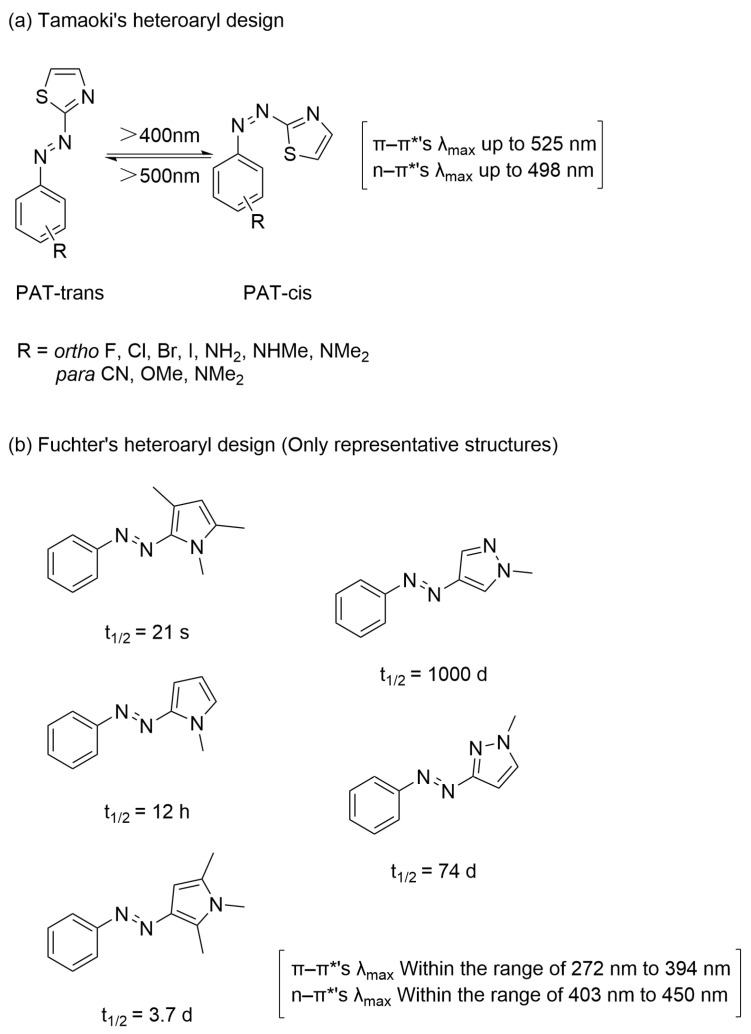
(**a**) Azobenzene-thiazole compounds’ substituent derivatization and near-infrared response characteristics. (**b**) Different aryl aziridine ring derivatives have specific effects on the response wavelength and thermal half-life.

The utilization of heteroaryl azobenzene dyes has also enabled the preparation of azobenzene derivatives exhibiting distinct photoresponsive characteristics [6,49,50,51,52]. Heterocyclic rings modulate the energies of π → π* and n → π* transitions through regulation of overall aromaticity, thereby altering the photoresponsive properties of the molecular system [53,54]. Tamaoki and co-workers reported the preparation of a series of novel benzyl azobenzene-thiazole compounds (Figure 6a) [9]. In these structures, one benzene ring was replaced by a thiazole moiety via regioselective cross-coupling, substantially modifying the electronic properties of the conjugated π-system. This alteration in molecular orbital energetics, particularly the n → π* transition energy, resulted in a redshift of the excitation wavelength. The system displays bidirectional photochromic behavior under visible light (wavelengths ≥ 400 nm for *trans → cis* conversion) and longer-wavelength visible light irradiation (wavelengths ≥ 500 nm for *cis → trans* conversion). Compared to conventional ultraviolet-activated azobenzene systems, it offers enhanced resolution while retaining biocompatibility. Fuchter and co-workers incorporated aryl aziridine rings into the azobenzene scaffold, resulting in a broadly tunable photoresponsive system whose thermal half-life spans from seconds to years (Figure 6b) [49].

### 2.3. Photoresponse of Bridged Azobenzene

**Figure 7:**
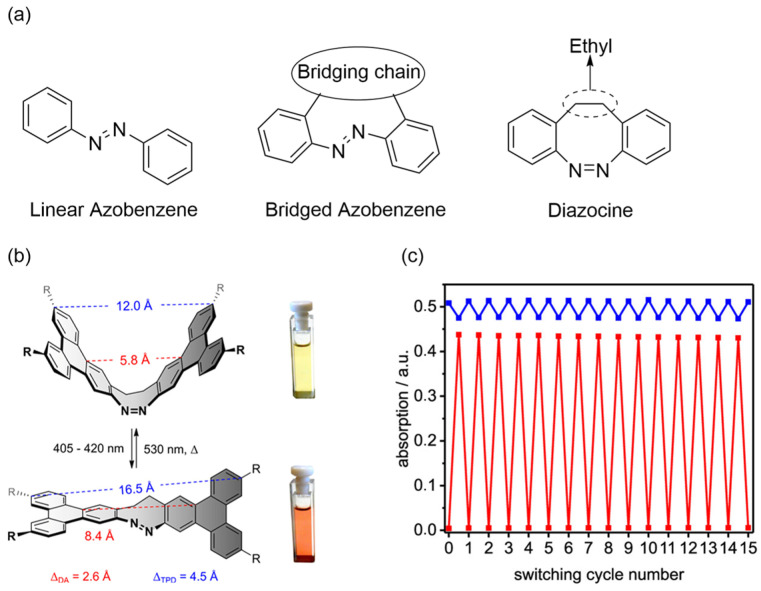
(**a**) Representative Structures of linear/bridged azobenzene and diazocine. (**b**) Photoisomerization of the triphenyl incorporated diazocine derivative induced by 405–420 nm and 530 nm irradiation. (**c**) The fatigue resistance test conducted under the irradiation cycles of 420 nm and 530 nm. (**b**,**c**) Reproduced with permission from Ref. [63]. Copyright (2025) American Chemical Society.

Bridged azobenzenes constitute a distinct subclass in which the aryl rings are linked not only by the azo bond but also by an additional covalent tether, forming a rigid tricyclic architecture (Figure 7a) [55,56,57]. Among these, diazocines stand out as the most prominent examples and feature an ethylene bridge (-CH_2_CH_2_-) at the *ortho* positions. This structural constraint inverts thermodynamic stability to favor the *cis* isomer and simultaneously enhances isomerization quantum yield, PSS ratios, and red-shifted absorption (405–530 nm) [58,59,60,61,62]. In 2025, Herges and co-workers reported the preparation of a triphenyl incorporated diazocine derivative (Figure 7b) [63]. As indicated previously, the system exhibits absorption bands at 405–420 nm and 530 nm, which are redshifted compared to those of conventional linear azobenzene. Additionally, the photostability of this compound was evaluated under cyclic irradiation with two excitation wavelengths, and robust fatigue resistance was demonstrated. (Figure 7c).

### 2.4. Photoresponse of Azobenzene Macrocycles

Azobenzene macrocycles represent another classical azobenzene. In general, the photophysical properties of azobenzene macrocycles are governed by ring size, molecular geometry, and the substitution patterns of peripheral substituents [64].

**Figure 8:**
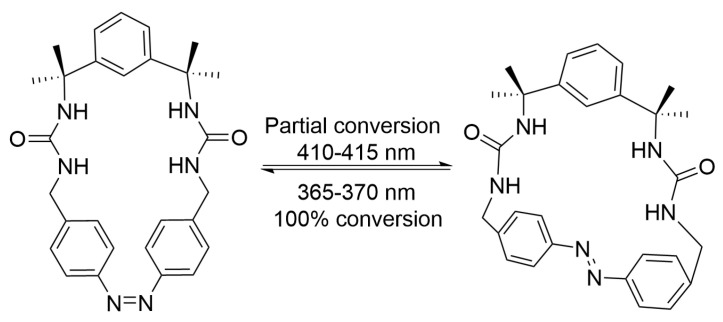
Interconversion between *trans* and *cis* isomer of azobenzene macrocycles.

In 2023, He and co-workers reported a novel azobenzene macrocycle with two urea moieties and one azobenzene unit, whose *cis* isomer shows higher thermodynamic stability than the *trans* isomer [65]. The authors attributed this stability inversion to the distorted macrocyclic framework, which stabilized the *cis* isomer and destabilizes the *trans* configuration. As shown in Figure 8, this system achieved quantitative (100%) unidirectional photoisomerization from *trans* to *cis* isomer, and exhibited excellent reversibility and fatigue resistance over multiple switching cycles.

**Figure 9:**
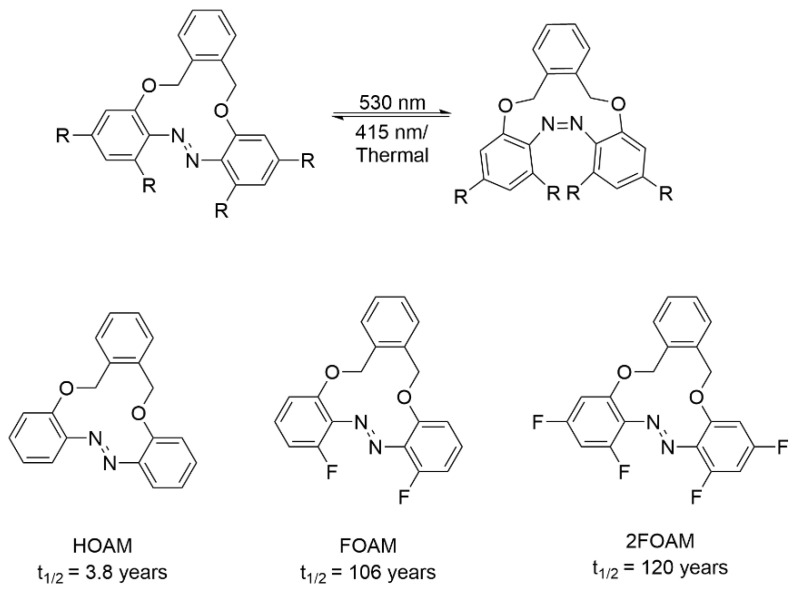
The structure of HOAM, FOAM, and 2FOAM.

In 2024, Staubitz’s group reported the synthesis of *ortho*-fluorinated 12-membered azobenzene macrocycles [31]. These compounds predominantly adopted the thermodynamically stable *trans* configuration, yet undergo efficient photoisomerization to the *cis* configuration upon 530 nm illumination. (Figure 9). Unlike linear azobenzenes, *ortho*-fluorination 12-membered azobenzene macrocycles extended the *cis* half-life to over 100 years at 25 °C without altering the excitation wavelength or *trans*/*cis* ratio of PSS.

In summary, azobenzene derivatives possess diverse structural types, and their photochemical properties and regulation mechanisms differ considerably with structural variation. This results in distinct applicability and application scenarios for different azobenzene architectures. Therefore, the rational selection and optimization of suitable azobenzene systems according to practical requirements represent a key prerequisite for the efficient and precise implementation of azobenzene-based photo-controlled functional systems.

## 3. Applications of Azobenzene in Medicinal Chemistry

Integrating photoresponsive azobenzene motifs into pharmacophores enables transformative light-regulated drug-receptor interactions. Researchers have advanced diverse pharmaceutical applications by leveraging tailored azobenzene scaffolds. This photopharmacological strategy achieves bidirectional efficacy modulation through the reversible spatial reorganization of azobenzene-based structures, thereby ensuring precise spatiotemporal control over drug activity and minimizing off-target effects.

A key conceptual framework in this endeavor is the use of privileged structures, molecular scaffolds with broad therapeutic relevance first defined by Ben Evans in 1988 [66,67]. Even for drug classes lacking innate light responsiveness, the incorporation of azobenzene units through these scaffolds provides a viable route to photoregulated pharmacophores. Structural integration is commonly achieved through covalent conjugation or bioisosteric replacement, with azologization, a methodology introduced by Trauner in 2014, serving as a targeted strategy for azobenzene incorporation that has been successfully applied across diverse compound classes [68].

This section highlights representative examples of photopharmacology to illustrate recent progress in the field, demonstrating how azobenzene-based molecular design continues to expand the toolbox for precision pharmacology.

### 3.1. Photopharmacological Applications of Neurological Drugs

Neurological drugs comprise therapeutic agents designed to modulate or restore function in the central and peripheral nervous systems. These compounds function through multiple mechanisms such as regulating neurotransmitter systems and ion channels, facilitating neural repair, and blocking pathological signal transmission [69,70]. The incorporation of photopharmacology into neurological drug development constitutes a promising approach, which improves the trade-off between therapeutic efficacy and safety profiles via precise spatiotemporal regulation of drug activity while minimizing adverse effects.

**Figure 10:**
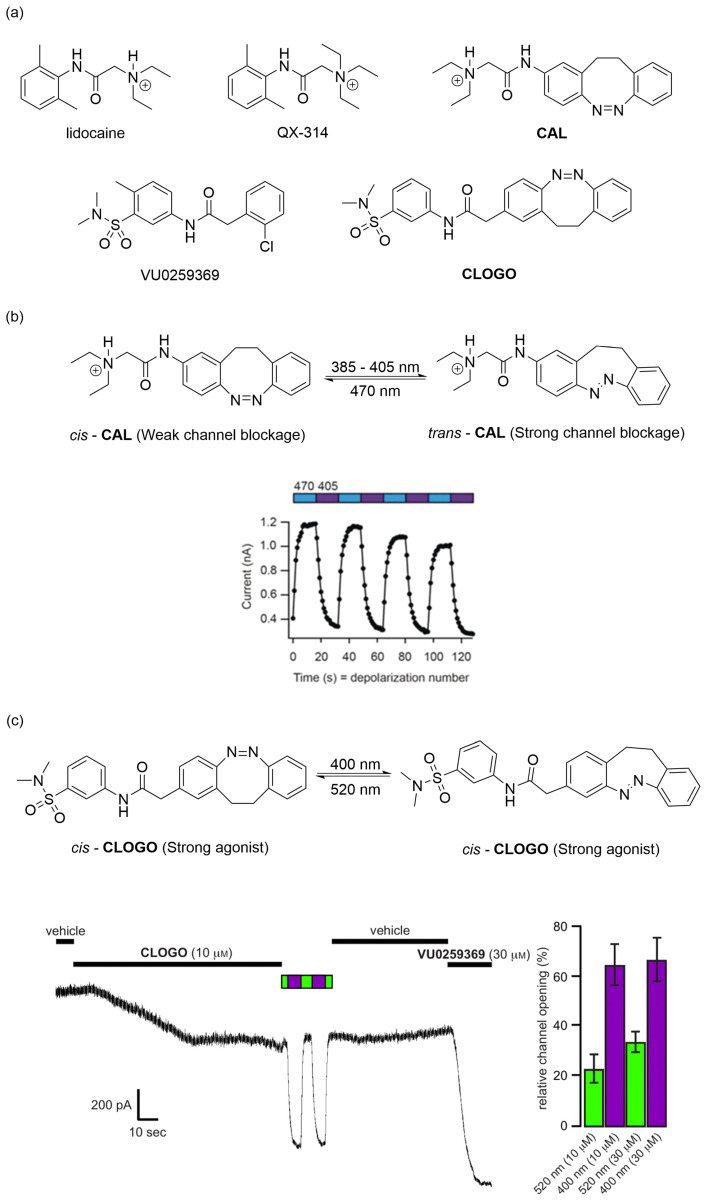
(**a**) Photoswitches of Kv and GIRK channels and their lead compound structures. (**b**) Assessment of CAL-induced photoswitchable blockade of Kv channels and its fatigue resistance. (**c**) Comparison of GIRK channels opening using the non-photoswitchable agonist VU0259369 (30 µM) and the *cis*- and *trans*-isomers of CLOGO (10 µM and 30 µM). (**b**,**c**) Reproduced with permission from Ref. [71]. Copyright (2019) Wiley Online Library.

In 2019, Trauner and co-workers leveraged the distinctive photoresponsive characteristics of the diazocine scaffold by incorporating it into ion channel-blocking active structures [71]. They developed a photochromic blocker CAL targeting voltage-gated potassium (Kv) channels and a photochromic activator CLOGO for G-protein-coupled inwardly rectifying potassium (GIRK) channels (Figure 10a). During the investigation of CAL, when the concentration reached 10 μM, a significant channel blockage phenomenon was observed, which was significantly attenuated under 470 nm illumination. The difference in blockade efficacy between light and dark conditions measured approximately 40.4%. Furthermore, based on the light-modulated current–voltage (I–V) relationship, the photoregulation of this antagonist was demonstrated to be reversible, which also demonstrated remarkable fatigue resistance of CAL. (Figure 10b). In GIRK channel activation assays, *cis*-CLOGO (10 μM) activated the channel to 23% of the level induced by the reference agonist VU0259369 (30 μM), whereas *trans*-CLOGO (10 μM) achieved 65% activation (Figure 10c). Doubling the *cis*-CLOGO concentration to 30 μM increased activation to 34%, while *trans*-CLOGO at 30 μM showed no significant further gain (68%), indicating saturation at 10 μM.

**Figure 11:**
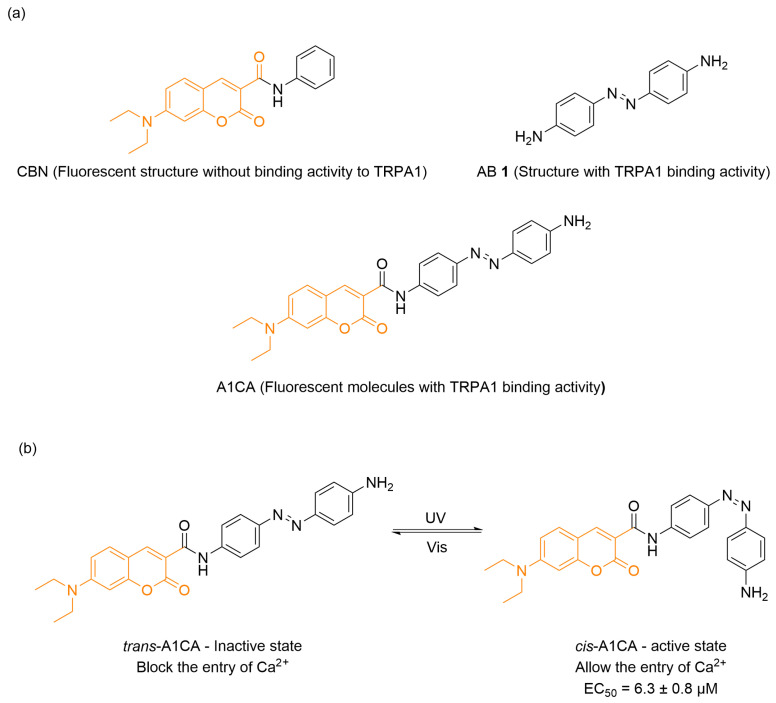
(**a**) Design strategy of TRPA1-targeting photochromic probe. (**b**) A1CA’s light regulation of TRPA1 channel activation.

In the same year, Zhang and co-workers introduced A1CA, a photochromic “turn-on” fluorescent probe targeting transient receptor potential ankyrin 1 (TRPA1) channel by combining a coumarin fluorophore CBN with an azobenzene derived AB **1** (Figure 11a) [72]. Fluorescence of the A1CA azobenzene-chromone complex was governed by solvent environment and intramolecular rotation. Upon glycerol addition to its aqueous solution, fluorescence intensity raised progressively with glycerol concentration due to the solvent’s high viscosity restricting azobenzene rotation. This restriction reduced non-radiative decay from the excited state, resulting in enhanced emission. The same principle applied when A1CA bound inside the channel protein: confined rotation under spatial constraint similarly led to fluorescence activation. Electrophysiological and confocal imaging assays demonstrated that A1CA exhibits binding-specific fluorescence at the TRPA1 channel but did not induce channel activation under dark conditions. On the contrary, A1CA specifically activated TRPA1 under UV illumination, with an EC_50_ of 6.3 μM, while showing minimal activity on other ion channels (Figure 11b). Thus, A1CA served as a potent photopharmacological tool, enabling simultaneous optical modulation and visualization of TRPA1-mediated pathways.

**Figure 12:**
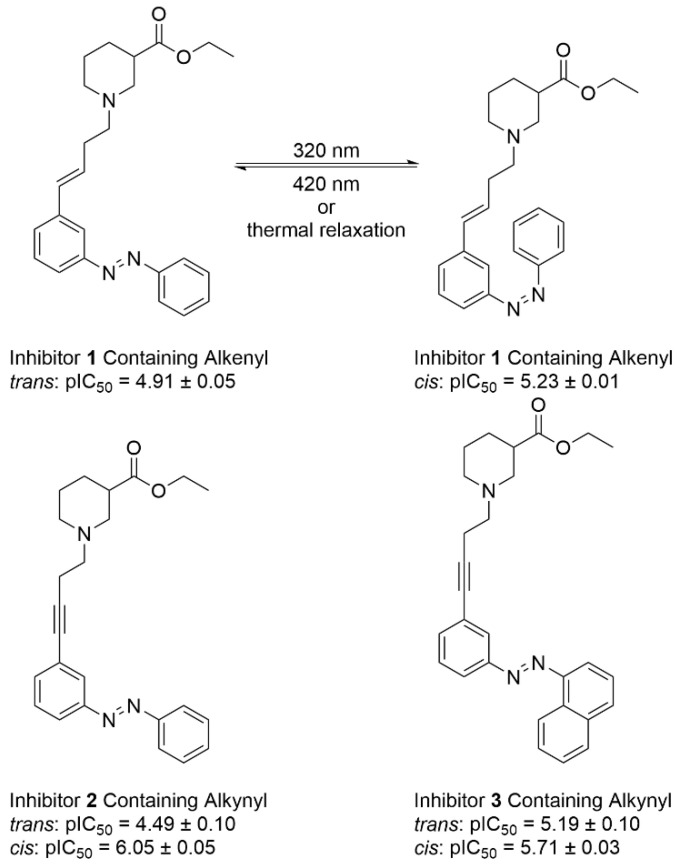
Azobenzene-based GAT1 inhibitors and the isomers exhibit marked differences in biological activity.

Wanner and co-workers reported the preparation of a series of azobenzene-substituted nicotinic acid derivatives (Figure 12) [73]. They further evaluated how the configuration of these compounds affected their inhibitory efficacy against γ-aminobutyric acid transporter subtype 1 (GAT1), which is the predominant GABA transporter isoform responsible for neurotransmitter reuptake in the central nervous system. This study established structure-activity correlations between azobenzene isomerization states (*trans/cis*) and GAT1 functional modulation capacity. Comprehensive pharmacological evaluation of geometric isomers revealed the inaugural identification of photoresponsive GAT1 modulators. The *trans*-configured analogues demonstrated intermediate inhibitory efficacy (pIC_50_ ranged from 4.49 to 5.19) against GABA uptake by murine GAT1 (mGAT1), whereas photoisomerization to the *cis*-configuration significantly enhanced inhibitory potency (pIC_50_ ranged from 5.23 to 6.05). This stereochemical activity correlation, attributable to photoisomerization-induced conformational reorganization of the azobenzene pharmacophore, exhibited remarkable selectivity across GABA transporter subtypes, establishing a foundational platform for optically controlled neurotransmitter regulation.

**Figure 13:**
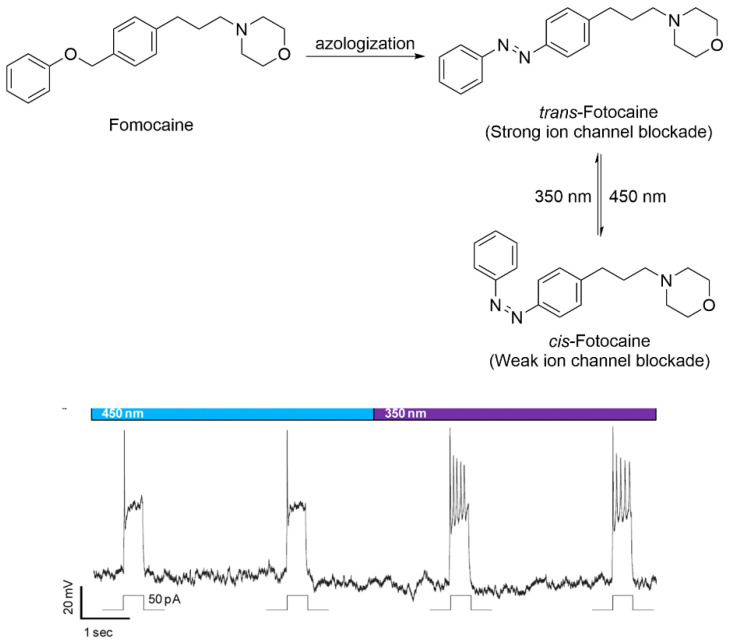
Development of ion channel blocker Fotocaine utilizing azologization strategy.

Fomocaine mainly functions by blocking voltage-gated ion channels. Trauner and co-workers utilized the azologization strategy to develop a unique ion channel blocker, Fotocaine, which exhibited significant photo-responsive activity (Figure 13) [68]. In the patch-clamp electrophysiological analysis of rat hippocampal neurons, Fotocaine (50 μM) exhibited reversible, light-gated regulation of hippocampal neuron activity: complete action potential (AP) suppression at 450 nm and restored firing at 350 nm, supporting its use as a photodynamic neuromodulator. This photoregulated bioactive structure demonstrated considerable potential as a photodynamic analgesic agent and represented a viable photopharmacological strategy for the optical control of pain.

**Figure 14:**
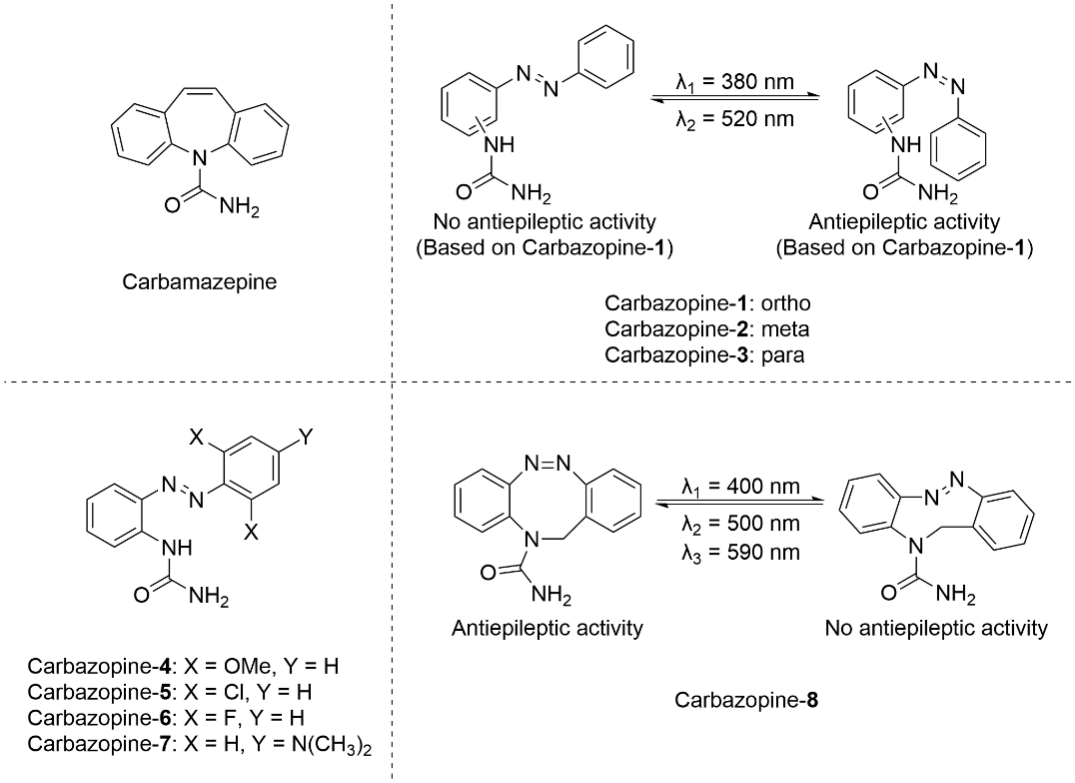
Series of Carbazopine derivatives and their photostimulated properties.

Gorostiza and co-workers designed a series of carbamazepine-derived azobenzene analogues featuring bridged tricyclic structures (Figure 14) [74]. The study introduced two compound sets: Carbazopine **1-3**, with urea groups substituted at various positions, and Carbazopine **4-7**, bearing different substituents on the benzene ring. Unlike previous studies that relied on conformational changes via azobenzene isomerization to mimic tricyclic structures, this work achieved a notable improvement by utilizing the diazocine derivative structure to construct the actual tricyclic structure. Two representative analogues, Carbazopine-**1** and Carbazopine-**8**, were selected for biological evaluation in a zebrafish model at 7 days post-fertilization, using swimming distance as a behavioral activity index. In the Carbazopine-**1** treatment group, exposure to UV light triggered *trans* → *cis* isomerization, yielding a tricyclic-like conformation. While UV light alone elicited strong avoidance behavior in untreated zebrafish (swimming distances exceeding 80 mm), treatment with Carbazopine-**1** resulted in dose-dependent inhibition of motility. At 100 μM, zebrafish exhibited near-complete unresponsiveness to UV stimulation, with swimming distances approaching zero. In contrast, zebrafish treated with Carbazopine-**8** remained immobile in darkness. Under 500 nm halogen light, locomotion was significantly suppressed, whereas 400 nm blue light induced isomerization, disrupting the tricyclic structure, and restoring activity, with swimming distances increasing to approximately 150 mm.

### 3.2. Photopharmacological Applications of Glycosidase Inhibitors

Glycosidases are found in nearly all living organisms and play crucial roles in numerous biological processes, including oligosaccharide synthesis, the formation of alkyl and aromatic glycosides, and the glycosylation of amino acids, polypeptides, and antibiotics [75,76,77,78]. The development of selective glycosidase inhibitors is highly important for advancing our understanding of glycan processing and for the discovery of new therapeutic agents. The photopharmacological research on glycosidase inhibitors provides an artificial regulatory space for the physiological processes regulated by this biological enzyme and offers an operational platform for precise treatment and targeted therapy.

**Figure 15:**
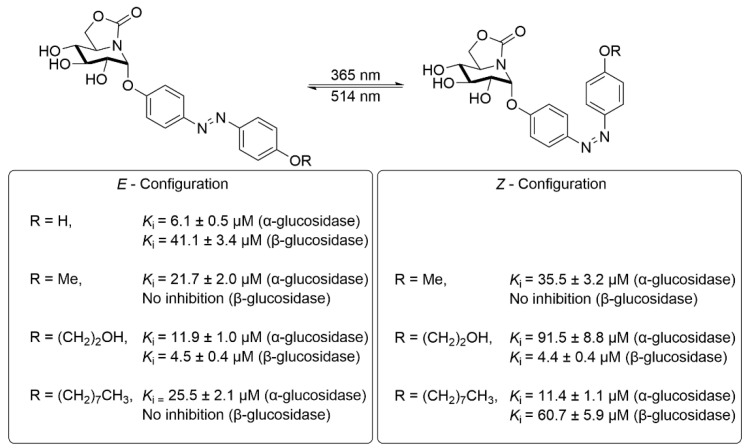
Photoswitchable sp^2^-iminosugar-azobenzene conjugates and their glucosidase inhibitory activity.

In 2024, Mellet’s group conjugated sugar-mimetic motifs with azobenzene photochromic units, obtaining *trans* and *cis* photoisomers that showed clearly differentiated glycosidase inhibition (Figure 15) [79]. They synthesized photoresponsible azobenzene α-glycosides using sp^2^-alkylamino sugars. Significant β-glucocerebrosidase inhibition was achieved by the *cis*-isomer, contrasting with weak *trans*-isomer activity. This configuration-specific inhibition enables controllable drug action via *cis*-isomerization, permitting optical activation and thermal reversion for programmable effects.

**Figure 16:**
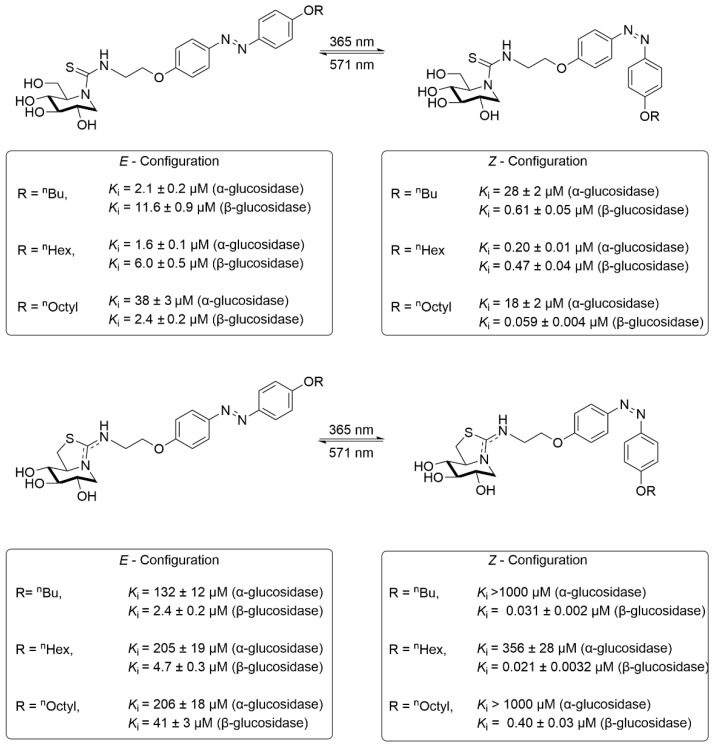
Optimized photoswitchable sp^2^-iminosugar-azobenzene conjugates and their glucosidase inhibitory activity.

In 2025, the same group extended the exploration of photoactivatable sp^2^-iminosugar derivatives (Figure 16), replacing the original sugar residue site with 1-deoxynojirimycin (DNJ) [80]. These DNJ-glycosylated photoresponsive inhibitors exhibited pronounced differences in their inhibitory activity against α-glucosidase versus β-glucosidase, often varying by orders of magnitude. Notably, upon switching from *trans* to *cis* configuration, their inhibitory potency toward β-glucosidase increased markedly, shifting from micromolar to nanomolar activity. For β-glucosidase, the sp^2^-iminosugar/azobenzene conjugates with a dual-ring structure displayed inhibition constants all below 1 μM, with switching factors varying from 0.03 to 0.0008. These results indicated that the *cis*-isomer invariably possessed higher inhibitory activity than the *trans*-isomer, exhibiting nanomolar-range inhibition constants and a potency enhancement exceeding 76- to 1250-fold.

### 3.3. Photopharmacological Regulation of Adrenergic Receptors

Adrenergic receptors are critical pharmaceutical targets, with their modulators widely used to treat diverse diseases such as cardiac arrhythmias, asthma, anxiety, and glaucoma [81,82,83,84]. Developing light-based methods to regulate β-adrenergic activity in a spatiotemporally precise manner thus offers significant scientific and therapeutic promise.

**Figure 17:**
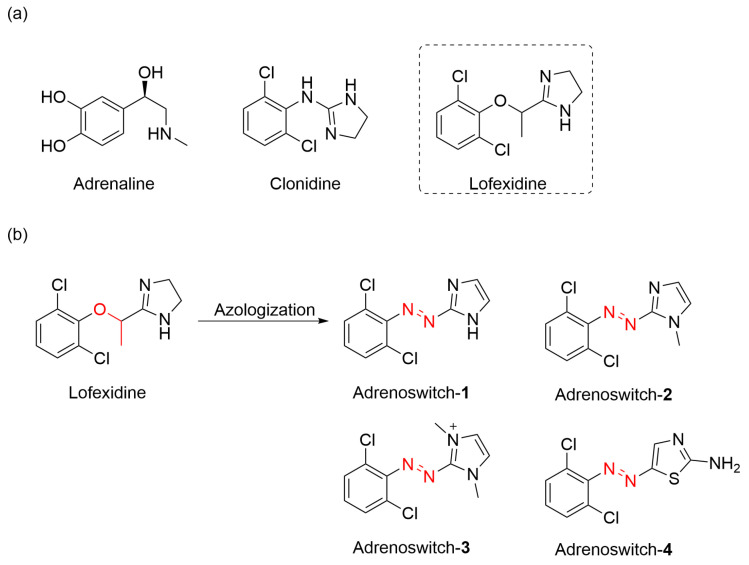
(**a**) The structure of adrenaline and some agonists of adrenaline receptors. (**b**) Rational design of Adrenoswitch **1**-**4** by azologization.

Building on established adrenergic ligands (Figure 17a), Gorostiza and co-workers engineered a series of photoresponsive molecular switches Adrenoswitch **1**-**4** capable of optical control over adrenergic signaling (Figure 17b) [85]. Competitive binding assays revealed that Adrenoswitches showed affinity for α_2_-adrenergic receptors in prefrontal cortex membranes comparable to Clonidine. In functional studies using isolated aortic rings, all Adrenoswitches exhibited significantly enhanced vasorelaxation upon UV illumination compared to Clonidine. Further evaluation in the zebrafish neurobehavioral model demonstrated the light-dependent activity of Adrenoswitch-**1** (50 μM), which progressively reduced larval motility under UV exposure, while Clonidine-treated groups remained unaffected by light conditions.

**Figure 18:**
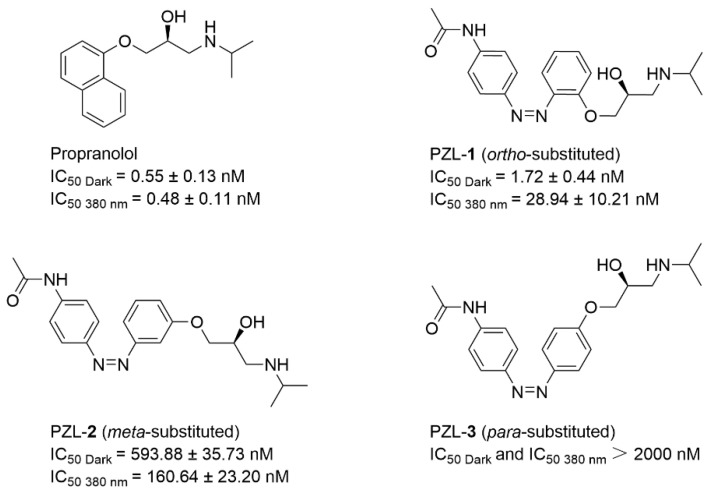
Photoswitchable azobenzene β_2_-AR antagonists photoazolols (PZLs).

In 2020, Rovira and co-workers developed a distinct class of light-responsive molecular switches targeting β_2_-adrenoceptors, in contrast to Gorostiza’s focus on other adrenergic receptor subtypes [86]. These azobenzene-based systems achieved fully reversible *trans ↔ cis* photoisomerization when alternately irradiated with 380 nm and 550 nm light (Figure 18). Among the three synthesized compounds, PZL-**1** and PZL-**2** functioned as effective photoregulated ligands, enabling reversible optical control over β_2_-AR-mediated cellular responses. Notably, these two compounds exhibited opposing photopharmacological profiles: PZL-**1** demonstrated significantly higher potency in the dark (IC_50_ = 1.72 nM) compared to its activity following 380 nm irradiation (IC_50_ = 28.94 nM). Conversely, PZL-**2** exhibited enhanced receptor inhibition upon light exposure (IC_50_ = 160.64 nM), representing a substantial increase in potency relative to its dark-adapted state (IC_50_ = 593.88 nM).

**Figure 19:**
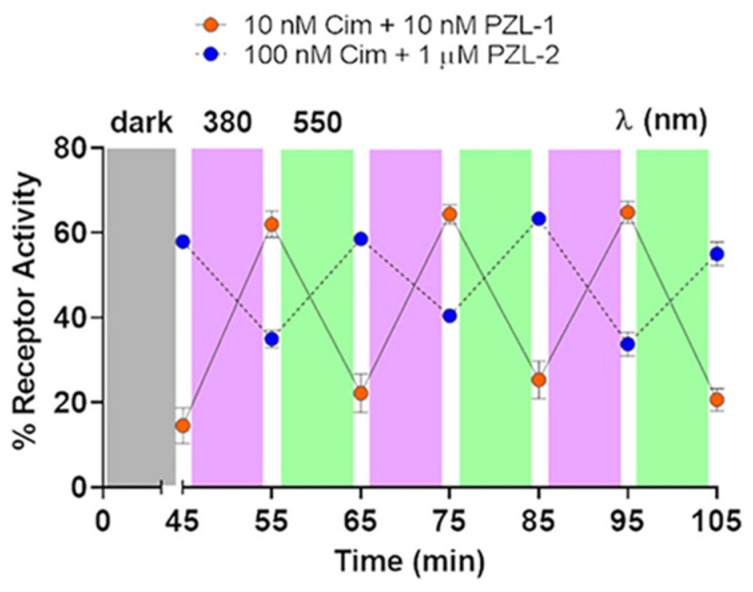
Time-course cAMP quantification in cimaterol-stimulated cells in the presence of PZL-**1** (orange dots) and PZL-**2** (blue dots). Reproduced with permission from Ref. [86]. Copyright (2020) American Chemical Society.

This study subsequently verified the photo-triggered reversibility of PZL-**1** and PZL-**2**. This verification considered whether light could disrupt the interaction between the ligand and the β_2_-receptor when the active isomer was bound. It confirmed reversible optical control of β_2_-adrenergic receptor activity using PZL-**1** and PZL-**2** over multiple switching cycles (Figure 19). Dark-adapted PZL-**1** nearly fully suppressed receptor activation, while violet light attenuated this inhibition—an effect reversed by green light. In contrast, PZL-**2** reduced receptor activity by approximately 30% under violet light, with complete reversion upon green illumination. This reproducible photoswitching demonstrates effective disruption of ligand-receptor interactions and substantial anti-fatigue performance. This investigation further elucidates drug-active molecular scaffolds possessing novel photopharmacological and photochemical attributes, thereby advancing the mechanistic understanding of β_2_-adrenergic receptor signal transduction.

**Figure 20:**
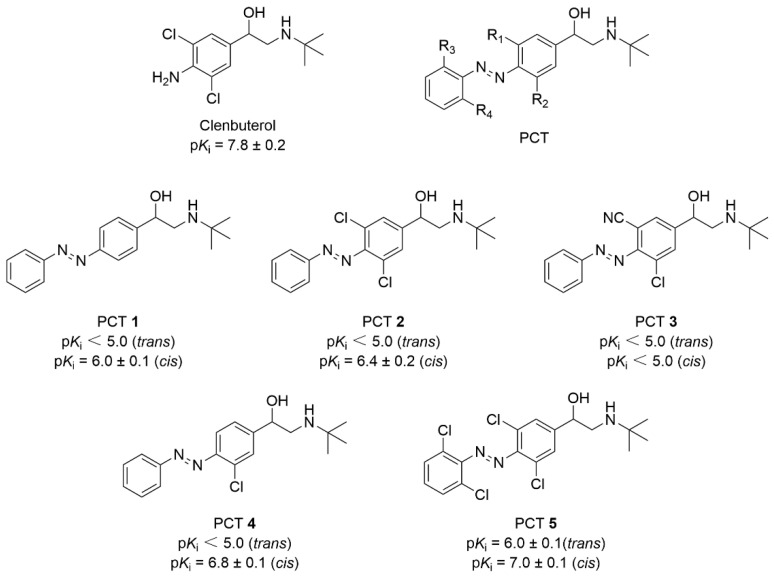
Structure of photoswitchable β_2_-AR agonists based on clenbuterol.

Wijtmans et al. developed a series of light-switchable β_2_ adrenergic receptor ligands (PCTs), based on the structure of the classic β_2_ adrenergic receptor agonist clenbuterol (Figure 20) [87]. PCTs in general demonstrated at least 25-fold increase in binding affinity upon *trans*−*cis* photoisomerization (Δp*K_i_* > 1.4). The introduction of a chlorine atom to the azobenzene structure was found to enhance binding affinity following photo-isomerization to the *cis*-form, as evidenced by comparative analysis of PCT **2**, **4**, and **5** with PCT **1**. Replacing with cyano groups (PCT **3**) would weaken the binding ability of the *trans* isomer and the *cis* state of PSS to the β_2_-AR, showing a significant difference compared to PCT **2**. In HEK293 cells, β_2_-adrenergic receptor activity was evaluated using agonists isoproterenol and clenbuterol. PCT **2** exhibited partial agonist activity with an intrinsic activity of 0.5 and pEC_50_ of 6.7 ± 0.3, while other PCTs showed no agonist effects. All PCTs inhibited isoproterenol-induced cAMP production upon *cis*-configuration activation. Although PCT 2′s agonist mechanism remains unknown, its reversible activation before and after stimulation provides complementary characteristics to existing photo-switchable β_2_-adrenergic receptor agonists.

### 3.4. Photopharmacological Applications of Cholinergic Drugs

Cholinesterases are serine hydrolases with vital functions in the central and peripheral nervous systems, a family which includes acetylcholinesterase (AChE, EC 3.1.1.7) and butyrylcholinesterase (BChE, EC 3.1.1.8) [88,89,90]. The development of cholinesterase-targeting and cholinergic receptors drugs is characterized by needs for high precision and strict safety. Photopharmacology presents a novel approach to address these challenges, utilizing light-sensitive compounds to achieve precise control of drug action and minimize adverse effects.

**Figure 21:**
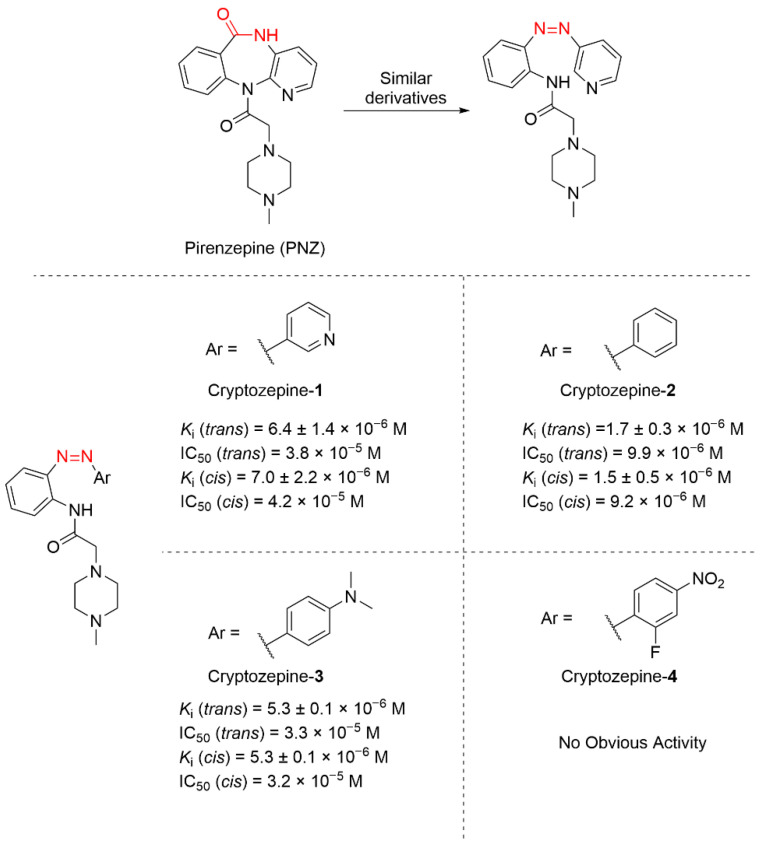
Cryptozepine azobenzene derivatives as photoswitchable M1R antagonists.

Based on the tricyclic pharmacophore of pirenzepine, the Gorostiza’s group established a photopharmacological approach using azobenzene-based derivatives (Figure 21) [91]. This molecular design preserved the structural topology critical for muscarinic acetylcholine receptor (M1R) antagonism while introducing bidirectional photonic control over receptor-ligand interactions. Systematic evaluation of equilibrium dissociation constants revealed Cryptozepine-**2** as the optimized ligand, with the lowest *K*_i_ and IC_50_, exhibiting superior M1R binding affinity and reversible light-dependent activity modulation. In vivo studies of this compound showed that mouse heart rates decreased from 360 beats/min (baseline) to 150 beats/min after CCh administration. Cryptozepine-**2** exhibited configuration-dependent activity: its *trans*-form showed no antagonism, however the *cis*-form illumination restored rates to 250 beats/min. This light-regulated mode could accurately activate the local therapeutic effect, thereby reducing side effects and systemic toxicity.

**Figure 22:**
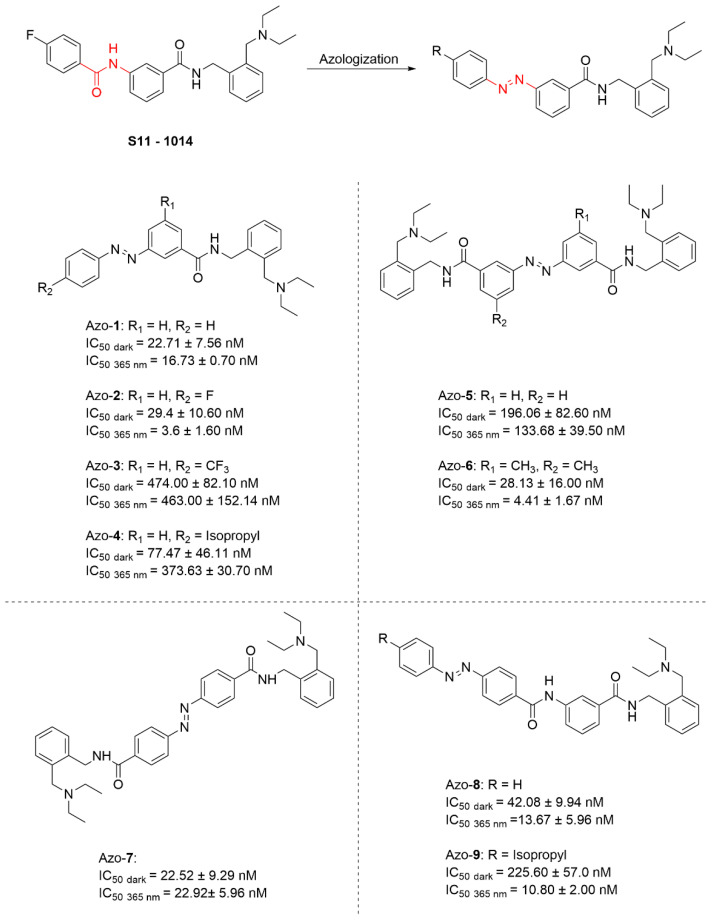
Design and pharmacological data of azobenzene-based photoswitchable BChE inhibitors.

Sun and co-workers pioneered the design of a new series of photoswitchable BChE inhibitors, which was achieved by replacing one of the amide bonds in the structure of S11-1014 with azo bond (Azo **1-7**) or by expanding the S11-1014′s scaffold through azo bonds (Azo **8-9**) (Figure 22) [92]. Notably, Azo-**4** exhibited a pronounced difference in efficacy between its *trans* and *cis* configurations, with an eightfold increase in activity observed upon photoisomerization. However, compound Azo-**5** exhibited reduced inhibitory potency against BChE in both isomeric forms, with only a marginal difference in activity between configurations. During subsequent structural optimization in Azo **8-9**, the core scaffold of S11-1014 was retained while incorporating the azobenzene unit as a structural extension. The optimal compound Azo-**9** was identified, exhibiting a more than 20-fold difference in activity between its *trans* and *cis* isomers.

**Figure 23:**
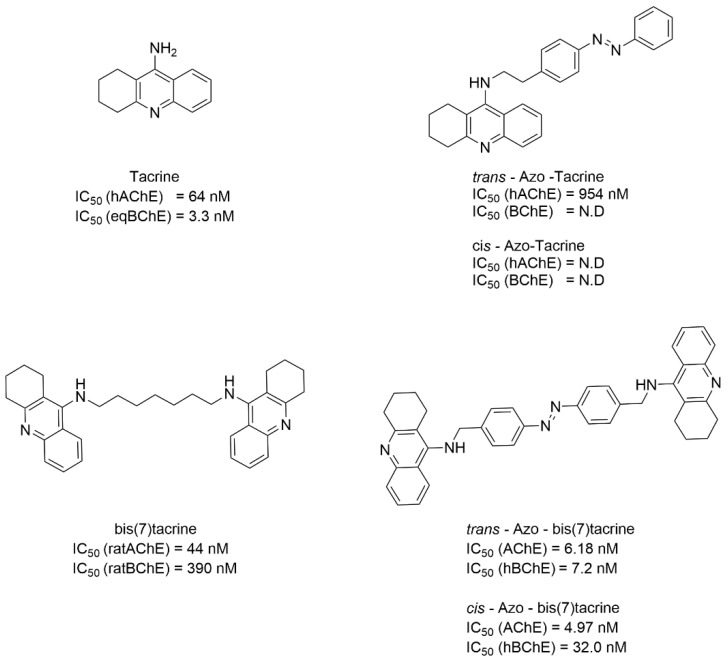
Structures of Tacrine, bis(7)tacrine, and novel azobenzene-based Tacrine derivatives.

Based on the known cholinesterase inhibitors Tacrine and bis(7)tacrine, Decker and co-workers developed a novel azobenzene-derived diester cholinesterase inhibitor Azo-tacrine and Azo-bis(7)tacrine, which exhibited more rapid inhibition compared to the template compound (Figure 23) [93]. Furthermore, azobenzene photoisomerization conferred light-dependent activity regulation to these compounds. Although no satisfactory results were obtained in the activity study of Azo-tacrine, Azo-bis(7)tacrine exhibited greater potency against hAChE (IC_50_ = 4.97–6.18 nM) compared to Tacrine (IC_50_ = 64 nM). While both configurations show similar selectivity for hAChE, light irradiation induced an approximately fivefold difference in inhibitory potency against the isoenzyme hBChE. In addition, reversible photoisomerization could be cycled multiple times without exhibiting optical fatigue, offering substantial advantages for advanced pharmacological applications.

### 3.5. Application of Photopharmacology in Cancer Treatment

The efficacy of cancer chemotherapy is often constrained by limited drug options and adverse effects. Identifying innovative solutions to improve selectivity and localization is therefore essential. Photopharmacology represents an innovative approach, where light-induced changes in pharmacological activity enable the development of precisely controlled anticancer drugs, showing considerable potential for advancing cancer treatment.

**Figure 24:**
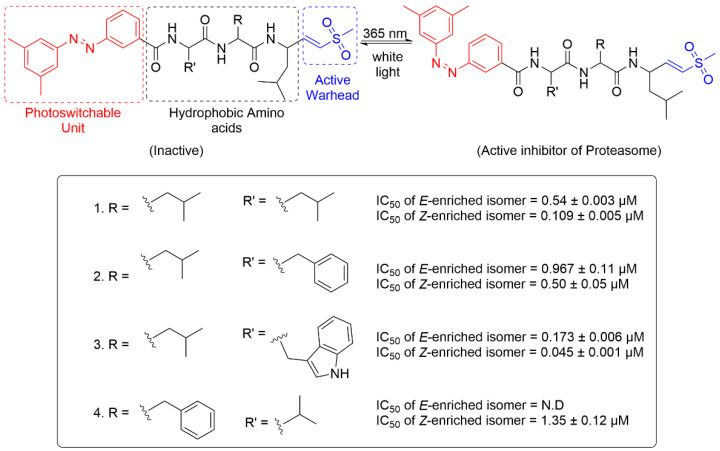
Design strategy used for developing Photopeptides **1**-**4**.

Precise regulation of proteasome activity represents a promising therapeutic strategy for cancer treatment. Thilagar and co-workers developed a novel series of azobenzene derived proteasome inhibitors (Figure 24) [33]. The molecular structure features a three-component design: an ethylene sulfone moiety serving as the covalent warhead for binding to the catalytic threonine residue in the proteasome active site; hydrophobic amino acid residues that enhance binding affinity by occupying the hydrophobic pocket of the enzyme; and an azobenzene unit that confers light-responsive activity to the molecule. Among all the analogues, Photopeptide **4** exhibited the most favorable PSS profile, demonstrating a marked activity difference upon 365 nm irradiation. During assessment of proteasome inhibition, prior to illumination, the compound showed negligible activity, whereas exposure to 365 nm light yielded an IC_50_ value of 1.35 μM, corresponding to a greater than 100-fold enhancement in inhibitory potency. Subsequently, the cytotoxicity of Photopeptide **4** on the HeLa, A549 and MCF-7 cell lines was evaluated. The fold change of *cis/trans* isomer in cell viability inhibition reached 3.3 in the HeLa cells, 19.3 in the MCF-7 cells, and 6.6 in the A549 cells. It was noteworthy that the fold change of this compound in the MCF-7 cell line was the largest, suggesting its potential to serve as a selective inhibitor for this specific cell type.

**Figure 25:**
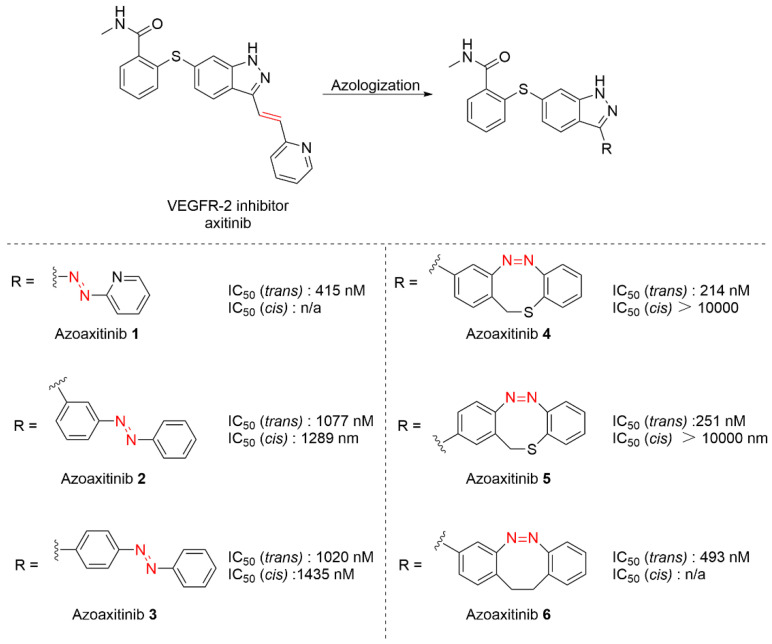
Axitinib’s azologization derivatives Azoaxitinib **1-6** and their biological activity.

The development of highly effective vascular endothelial growth factor receptor 2 (VEGFR2) inhibitors remains one of the persistent research priorities in oncological therapeutics. This focus is driven by the established clinical strategy of VEGFR2-based anti-angiogenic therapy for solid tumors. The Peifer’s group employed axitinib as a molecular template, incorporating linear and bridged azobenzene moieties as photoresponsive elements, thereby enabling the development of reversibly photoswitchable inhibitors targeting VEGFR-2 (Figure 25) [94]. Different design strategies were applied for the preparation of target molecules. The most straightforward method was to replace the C=C bond in axitinib with N=N bond, affording the most similar analogue Azoaxitinib **1**. For Azoaxitinib **2**-**6**, attempts were made to integrate various azobenzene or diazocine moieties, respectively. During biological evaluation, the highest inhibitory activity was shown by the *trans* conformation of Azoaxitinib **6**, while no significant activity was detected for its *cis* form. Furthermore, the incorporation of the dibenzothiophenone moiety led to markedly greater activity under photoexcitation compared to linear azobenzene analogues. This enhanced photoresponsiveness better meets the design objectives of photodynamic therapy.

**Figure 26:**
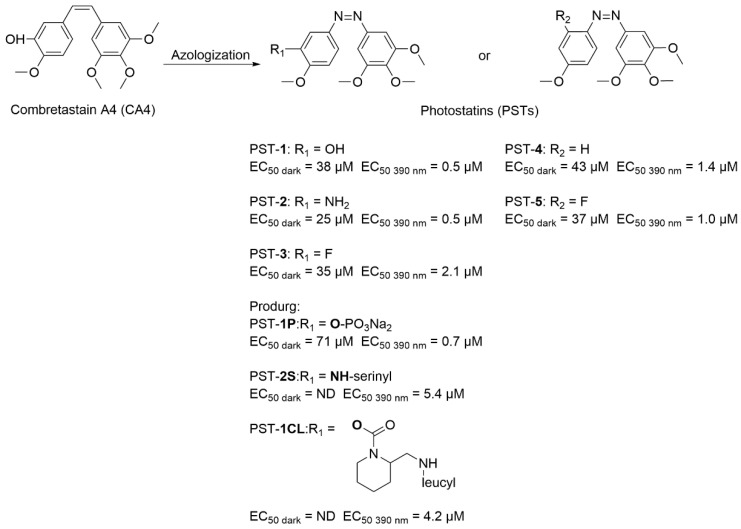
Structures of photochromic combretastatin A-4 analogs (PSTs) and their light-dependent cytotoxicity in MDA-MB-231 and HeLa cells.

Although compounds that interfere with microtubule dynamics are clinically employed as anticancer drugs, their lack of cellular specificity frequently causes severe side effects, thereby limiting treatment outcomes [95,96,97]. Thorn-Seshold and co-workers developed a novel class of reversible photochromic molecules PSTs through structural modification of combretastatin A-4 (CA4) via azobenzene incorporation (Figure 26) [98]. Cytotoxicity evaluation in MDA-MB-231 human breast cancer cells demonstrated that light irradiation induced potent cytotoxic effects (EC_50_: 0.5–5.4 μM), whereas minimal toxicity was observed under dark conditions (EC_50_ > 25 μM). This distinct light-dependent activity profile confirmed the precise controllability of PSTs’ bioactivation. Among these compounds, PST-**1CL** was identified as the optimal. It showed almost no activity under dark conditions, which was consistent with PST-**2S**. However, after being irradiated by 390 nm light, the activity of PST-**1CL** (EC_50_ = 4.2 μM) was superior to that of PST-**2S** (EC_50_ = 5.4 μM). This was in line with the design for ensuring medication safety control. Consistent results were obtained in HeLa cervical cancer cells, further validating the reproducible photoregulation of cytotoxicity across different cancer models.

Proteolysis targeting chimeras (PROTACs) are heterobifunctional degraders comprising two ligands connected by a linker: one that binds to the target protein and the other that recruits E3 ubiquitin ligase [99,100,101,102]. This approach utilizes the ubiquitin-proteasome system to selectively degrade disease-related proteins and represents a major research focus in clinical oncology. However, their distinct mechanism of action presents potential risks during systemic administration, which may affect both cancerous and normal tissues. One of the methods that can enhance the selectivity of drugs is to incorporate light-responsive structures, using light to regulate the activity [99,103,104].

**Figure 27:**
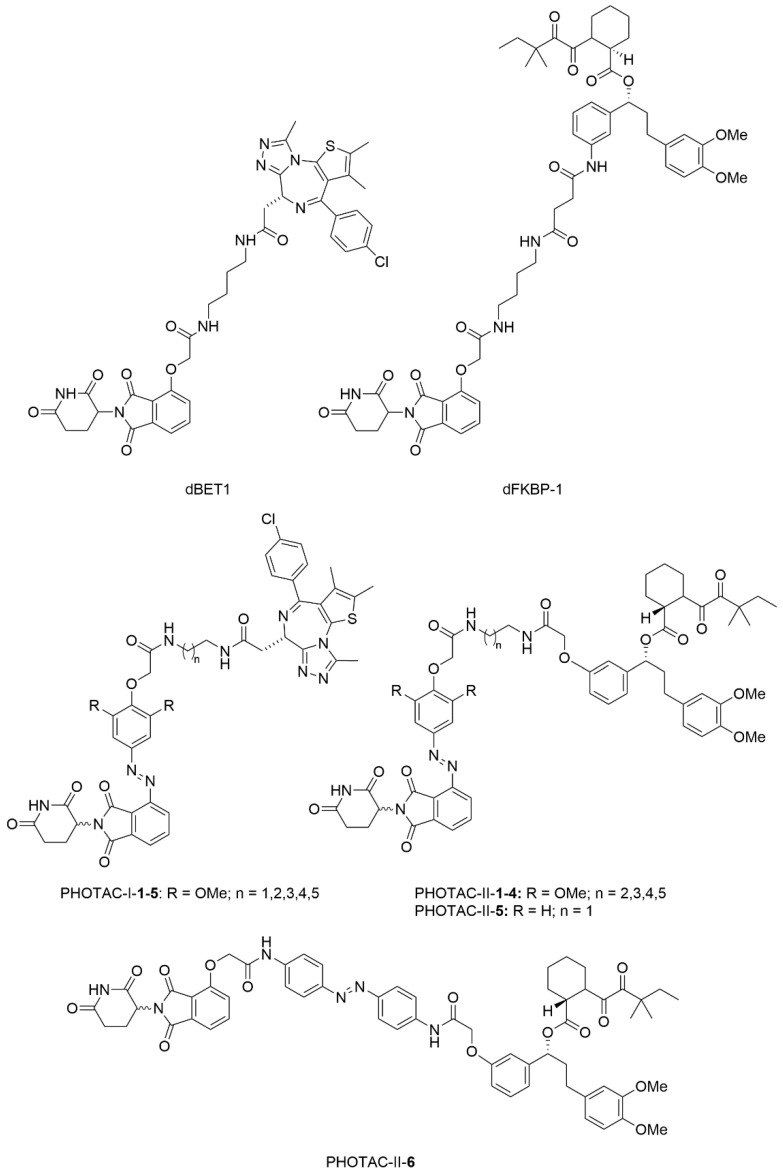
Structures of PROTACs dBET1, dFKBP-1, and representative members of PHOTAC-I and PHOTAC-II series.

In 2020, Trauner and co-workers incorporated azobenzene into PROTAC structures, developing light-responsive molecules termed PHOTACs (PHOtochemically TArgeting Chimeras) (Figure 27) [99]. Based on the PROTAC dBET1 targeting BRD proteins, PHOTAC-I series was developed. These molecules showed minimal activity in darkness but could be activated by blue-violet light (380–440 nm) to degrade target proteins. In viability assays with RS4;11 cells under 390 nm light, PHOTAC-I-**3** performed the best, with an EC_50_ of 88.5 nM compared to 631 nM in the dark. Western blot analysis confirmed light-dependent degradation: PHOTAC-I-**3** strongly reduced BRD3 and BRD4 levels when illuminated, with only moderate effects on BRD2. No degradation occurred in the dark. To demonstrate broader applicability, PHOTAC-II was synthesized based on dFKBP-1, which targets the prolyl *cis*-*trans* isomerase FKBP12. PHOTAC-II-**5** and PHOTAC-II-**6** were proved to be the most effective. Under 390 nm light, PHOTAC-II-**5** largely eliminated FKBP12 within 6–12 h, while no degradation was observed in the dark. PHOTAC-II-**6** behaved similarly but showed more dark activity at the 24-h time point. These results validate PHOTACs as a versatile platform for optically controlled protein degradation with potential for biological research and clinical use.

## 4. Conclusions

Azobenzene-based molecular switches constitute a robust and versatile platform for the optical control of molecular structure and function. Their reversible *trans ↔ cis* photoisomerization enables precise, non-invasive regulation of geometry, dipole moment, and electronic properties through wavelength-specific light irradiation. Advances in molecular design, including strategic *ortho*-functionalization, conjugation extension, and substitution patterning, have significantly enhanced photoisomerization efficiency, thermal stability, and PSS distribution. Notably, the expansion of activation wavelengths from ultraviolet to visible and near-infrared regions has substantially broadened the biomedical applicability of azobenzene derivatives. Particularly, photoisomerization-induced alterations in structure–activity relationships often lead to marked differences in pharmacological efficacy, facilitating the integration of azobenzene motifs into diverse biologically active systems and advancing the fields of photopharmacology and photodynamic therapy.

Despite these achievements, several challenges remain, including incomplete photoconversion, limited fatigue resistance, and suboptimal thermal persistence of the isomer after irradiation. Addressing these limitations will require continued innovation in rational molecular engineering and computational design, coupled with the incorporation of orthogonal stimuli to achieve multi-level control. Promisingly, current research is steadily progressing toward solving these issues. The distinct photoresponsive properties of linear azobenzene and diazocine allow for flexible application-specific selection, and structural engineering strategies continue to improve their operational durability. Owing to their unique photochemical characteristics and structural tunability, azobenzene derivatives remain at the forefront of responsive molecular technologies. With ongoing developments, they hold strong potential for transformative applications in smart therapeutics, programmable nanomaterials, and molecular-scale devices.

## Data Availability

Data is contained within the article.

## Abbreviations

The following abbreviations are used in this manuscript:

PSS	Photostationary state
UV-Vis	Ultraviolet-visible
t_1/2_	Half-life
DFT	Density functional theory
GIRK	G-protein-coupled inwardly rectifying potassium
TRPA1	Transient receptor potential ankyrin 1
GAT1	γ-aminobutyric acid transporter subtype 1
AP	Action potential
I–V	Light-modulated current–voltage
DNJ	1-deoxynojirimycin
PCT	Light-switchable β_2_ adrenergic receptor ligand
AChE	Acetylcholinesterase
BChE	Butyrylcholinesterase
M1R	Muscarinic acetylcholine receptor
PZL	Photoazolol
VEGFR2	Vascular endothelial growth factor receptor 2
PROTAC	Proteolysis targeting chimera
PHOTAC	PHOtochemically TArgeting Chimeras
